# Distinct laminar origins of sensory-evoked high-gamma and low-frequency ECoG signals revealed by optogenetics

**DOI:** 10.1073/pnas.2516293123

**Published:** 2026-04-01

**Authors:** Pierre-Marie Garderes, Daniel E. Feldman, Kristofer E. Bouchard

**Affiliations:** ^a^Department of Neuroscience, University of California, Berkeley, CA 94720; ^b^Helen Wills Neuroscience Institute, University of California, Berkeley, CA 94720; ^c^Biological Systems and Engineering Division, Lawrence Berkeley National Lab, Berkeley, CA 94720; ^d^Redwood Center for Theoretical Neuroscience, University of California, Berkeley, CA 94720; ^e^Scientific Data Division, Lawrence Berkeley National Lab, Berkeley, CA 94720

**Keywords:** electrocorticography, high-gamma, laminar origin, inverse problem, optogenetics

## Abstract

Electrocorticography (ECoG) provides a high-spatiotemporal-resolution measure of cortical activity (cortical surface electrical potentials, CSEPs) in humans and animals. The CSEP high-gamma band (Hγ, 65 to 170 Hz) correlates with neuronal firing rates at the columnar spatial scale and is widely used as a biomarker of local activity. Whether Hγ reports all stages of columnar processing, intermediate processing in L2/3 (close to the ECoG electrode), or the main columnar output in L5, is unknown. We disentangled the laminar origins of Hγ and other ECoG bands by optogenetically suppressing L2/3 or L5 pyramidal cells during micro-ECoG recording in the mouse somatosensory cortex. Whisker deflections evoked transient, topographically localized CSEPs. L5 optogenetic suppression most strongly reduced 65 to 450 Hz (Hγ-uHγ) bands in sensory-evoked ECoG signals, whereas L2/3 suppression most strongly reduced 4 to 27 Hz (θ-β) bands. Thus, different CSEP frequency bands reflect layer-specific activity and are biomarkers of distinct stages of sensory-evoked columnar processing.

## Significance

Electrocorticography (ECoG) is a widely used measure of cortical activity in humans and animals, providing a unique methodological bridge from basic neuroscience discovery to understanding the human brain in health and disease. However, the cell types and laminar sources that generate ECoG signals are unknown, impeding interpretation of ECoG findings in both clinical and basic research. We disentangled the laminar origins of sensory-evoked ECoG frequency bands by optogenetically suppressing L2/3 or L5 pyramidal cells during ECoG recording in the mouse somatosensory cortex. We found that L5 optogenetic suppression most strongly reduced high frequencies whereas L2/3 suppression most strongly reduced lower frequencies. Thus, different ECoG frequency bands reflect layer-specific activity and are biomarkers of distinct stages of sensory-evoked columnar processing.

Electrocorticography (ECoG) is widely used as a mesoscale measure of cortical activity in human clinical settings and for basic research in animals (1–10). In micro-ECoG (µECoG), small, closely spaced surface electrodes measure cortical surface electrical potentials (CSEPs) with high spatiotemporal resolution (11). CSEPs contain both periodic and aperiodic signal components with energy across multiple frequencies (12). Among these, high gamma (Hγ: 65 to 170 Hz) is widely used as a signature of spatially localized neuronal activity in both humans (1, 4, 5, 7, 13–16) and animals (6, 9, 14, 17–21). However, the cell types and laminar sources that generate Hγ and other frequency components of CSEPs are unknown, which impedes interpretation of ECoG findings in both the clinic and basic research.

How different layers and cell types contribute to CSEP magnitude and frequency depends on biophysical factors for each cell type including distance to the surface electrode, number of cells of a given type, size, and alignment of each cell’s electrical dipole, and population synchrony (2, 22–24). CSEPs originate from a linear superposition of all transmembrane currents within a volume underneath the electrode (2). CSEPs are typically examined for their frequency content. Broadly speaking, low frequencies (4 to 30 Hz) are primarily thought to reflect summed synaptic potentials that are the input to neuronal populations (2, 24), higher frequency ranges (particularly Hγ) are often thought to reflect population spike rate across all neurons in the volume beneath the electrode (8, 9, 14, 18, 25, 26), while very high frequencies, i.e. multi-unit activity (MUA, 500 to 1,500 Hz) likely reflect the shape of spike waveforms themselves. However, whether high-frequency CSEPs reflect the firing of all neurons in the entire underlying cortical column, or specific neuronal populations in specific layers, is vigorously debated (14, 17). In the canonical cortical microcircuit, feedforward, event-related information is processed across cortical layers in sequence from layer 4 (L4) (the thalamo-recipient input layer in the sensory cortex) to L2/3 and then to L5, which is the major source of descending columnar output. If different CSEP frequency bands map to specific layers and cellular signals, these distinct CSEP bands could be used as biomarkers of columnar processing steps, providing a powerful approach to dissect cortical computations (14, 18, 26).

A recent study used simulations of a biophysically detailed model of a single cortical column from somatosensory (S1) cortex (27) to predict the laminar contributions to different frequency bands within sensory-evoked CSEPs (17). Activating thalamocortical inputs within the model evoked a simulated ECoG signal that quantitatively resembled actual sensory-evoked CSEPs from primary sensory areas. Numerical estimation within the model indicated that total transmembrane currents in L5, originating from all cell types and neuronal segments in the anatomical boundaries of that layer, contributed most of the high-gamma and ultra-high-gamma frequency (70 to 450 Hz, Hγ-uHγ) signal in the CSEP. In contrast, currents generated by all segments of all cell types in L2/3 contributed substantially less, despite being closer to the surface electrode. Importantly, the impact of L5 segments on CSEPs was assessed without altering currents from the remaining dendritic arbors or altering the large dipoles of these cells. Because most neuronal segments in L5 are L5 pyramidal (PYR) cell somas, proximal dendrites, and axons, this study predicts, surprisingly, that L5 PYR spiking is the largest source of Hγ in sensory-evoked CSEPs. If this is true, Hγ would reflect the final L5 output of each cortical column, while L2/3 activity (which represents an intermediate stage of columnar processing), could be more associated with a different CSEP frequency band.

Whether this preferential L5 origin of Hγ is true in vivo is unknown, in part because the prior simulation is single-column and thus lacks cross-columnar connections to L2/3 and other layers. Experimental studies that have sought to correlate CSEP frequency bands with neural activity in different layers have produced highly divergent findings, including that high-frequency CSEPs correlate with neural activity across all cortical layers (8); that CSEPs mostly reflect current-source density in superficial layers (14, 28); that intracortical gamma, high gamma, and spiking are dissociable (14, 25), and that L5 dendritic Ca2+ spikes and L1 single-unit action potentials may be observed in CSEPs (11, 29). Thus, the laminar origin of CSEP frequency bands remains fundamentally unresolved.

We took a different, causal approach using in vivo optogenetics to test whether L2/3 and L5 PYR neuron spiking contribute differentially to Hγ and other CSEP frequency bands. We recorded whisker-evoked CSEPs from mouse S1 with high-density µECoG arrays. We used the inhibitory opsin stGtACR2 (30) to optogenetically suppress spiking of either L2/3 or L5 PYR cells, using Drd3-Cre and Rbp4-Cre mice, which target L2/3 and L5 PYR cells, respectively (31–33). We found clear evidence that Hγ and uHγ bands in whisker-evoked CSEPs predominantly originate from L5 activity, while low-frequency (θ and ß) bands preferentially originate from L2/3. Thus, different frequency components of CSEPs have distinct laminar contributions, with Hγ and uHγ being dominated by columnar output from L5. The finding that different CSEP frequency components provide biomarkers of specific laminar activity could greatly enhance the interpretability of ECoG in a range of clinical and basic research settings.

## Results

### High-Gamma ECoG Activity in Whisker S1 Is Somatotopically Organized.

**Fig. 1.**
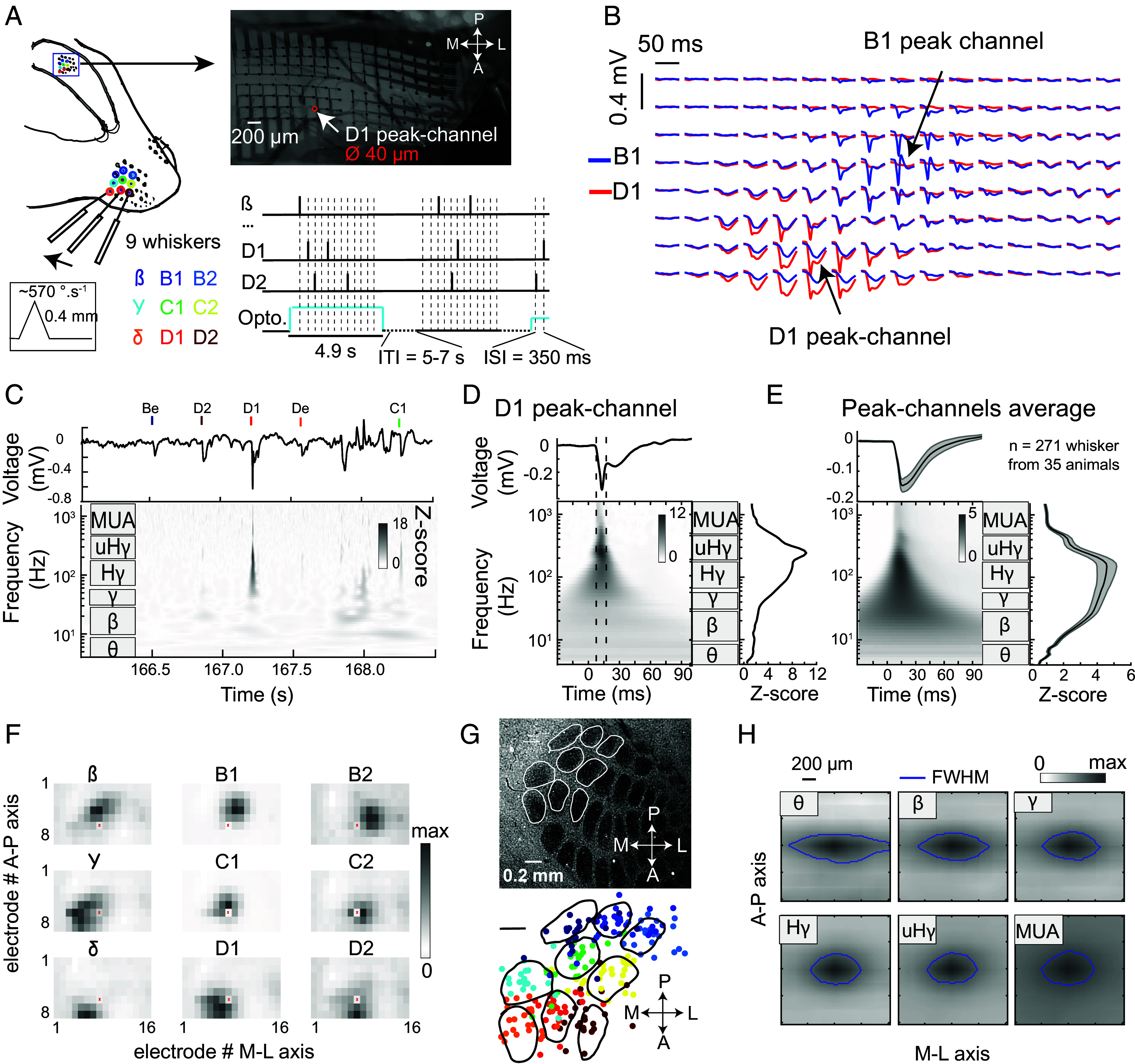
High-gamma ECoG activity in whisker S1 is somatotopically organized (*A*), Experimental approach, showing the 8 × 16 µECoG grid on the surface of S1 (pitch, 200 µm; electrode diameter, 40 µm) while whisker deflections are applied on nine contralateral whiskers. (*Lower* *Left*) Whisker deflection amplitude and velocity. (*Bottom* *Right*) Two illustrative trials showing optogenetic light and whisker stimuli (at dashed lines, chosen randomly among the nine whiskers). (*B*): Mean voltage traces for all 128 electrodes for stimulation of whisker B1 (blue) or D1 (red), for one recording session. Stimulus starts at 10 ms in each trace. (*C*), Raw voltage trace and time-frequency representation (z-scored to baseline epoch) in one portion of an example trial, recorded on the D1 peak-channel. (*D*), Mean D1 whisker-evoked activity on the D1 peak-channel from one experiment. (*Right*) Mean evoked activity in the frequency domain, measured in the response window (dashes). (*E*), Grand averaged evoked activity across all n = 271 responsive peak-channels for their columnar whisker, from 35 recording sessions. Conventions as in (*D*). Error shading is 95% CI. (*F*), Somatotopic structure of Hγ activity (z-score relative to baseline) on the ECoG grid for one example recording. Each subpanel is activity evoked by the labeled whisker. Each pixel is one electrode within the grid. The red dot is a defective channel. (*G*), Relative spatial location of whisker-evoked Hγ peaks on the ECoG grid. (*Top*) Column boundaries from cytochrome oxidase stain for one example mouse. *Bottom*, location of high gamma peak for each of the nine color coded whiskers from all sessions. Same color code as in (*A*). Eight points that were spatial outliers are omitted (see all points and details in *SI Appendix*, Fig. S1). Barrel outlines from the example case are overlaid for illustration, to scale and manually aligned in X-Y. (*H*), Mean spatial extent of whisker evoked activity on the grid (grayscale), separated by frequency band. Responses are aligned around the most responsive electrode in the indicated frequency band and interpolated at 10× resolution. The blue line is full width at half max (FWHM).

To characterize sensory-evoked CSEPs in mouse S1, we recorded with high-density µECoG arrays in head-fixed, lightly anesthetized Drd3-Cre or Rbp4-Cre mice as nine whiskers were independently deflected in a 3 × 3 array (Fig. 1*A*) (34). For each µECoG channel (Fig. 1*B*), we used wavelet decomposition to obtain the time-frequency representation of the evoked CSEP (Fig. 1 *C–E*). Stimulus-evoked power in each wavelet-defined frequency was z-scored to the prestimulus baseline, which enables evoked activity to be shown clearly relative to the inherent ~1/f^a^ power distribution of the electrophysiology signal. We annotate six physiologically relevant frequency bands: θ (4 to 8 Hz), ß (10 to 27 Hz), γ (30 to 57 Hz), Hγ (65 to 170 Hz), uHγ (190 to 450 Hz), and MUA (500 to 1,500 Hz) (2, 17). Mice expressed the inhibitory opsin stGtACR2 in either L2/3 PYR cells, L5 PYR cells, or not at all (see below).

We first analyzed the topographic organization of evoked CSEPs within whisker S1, examining trials with no optogenetic light. Single whisker stimuli evoked sensory responses at a localized subset of ECoG channels in S1 (Fig. 1*B*). For each whisker, we identified the ECoG channel with the strongest evoked response, termed that whisker’s peak-channel (Fig. 1*B* and *Methods*). Only peak-channels with substantial responses were analyzed (n = 271/324; average Z-score > 1). On average, sensory-evoked responses had power in all frequency bands, with the strongest response in Hγ, peaking 15 ± 0.2 ms (mean ± SEM) poststimulus, consistent with the known latency of spiking responses in S1 (10 to 30 ms across layers) (35, 36). Subsequent analysis of effects across frequencies at a specific time examined CSEPs within a ±5 ms window around the peak of the evoked response (e.g., Fig. 1 *D* and *E*).

Whisker-evoked Hγ CSEP signals were somatotopically organized in S1 and aligned with the known whisker map (Fig. 1 *F–H*). Each whisker evoked Hγ activity in a local cluster of electrodes whose location varied with whisker identity (see example in Fig. 1*F*). When peak Hγ channel location was localized relative to whisker barrel boundaries (obtained from post hoc cytochrome oxidase staining), the majority of peak responses (n = 128/225, localization from 25/36 sessions that included the identical set of nine whiskers) were located within the column boundaries for the corresponding whisker (Fig. 1*G*, see *SI Appendix*, Fig. S1 for the localization of all peak-channels). To estimate spatial spread of responses we calculated the mean CSEP activity centered on the peak-channel for each frequency band. The smallest spread was found in the Hγ (0.43 ± 0.01 mm^2^, mean area at half maximum ± SEM) and uHγ bands (0.44 ± 0.03 mm^2^). This corresponds to a circular radius of 370 µm, similar to the radius of whisker-evoked spiking in L2/3 PYR cells from two-photon calcium imaging (~350 µm) (37). Spread was significantly broader in the θ, ß, and MUA bands (Fig. 1*H*, *P* < 0.01, χ^2^ = 123.6, n = 271 peak-channels, Friedman test, see detailed post hoc comparisons in *SI Appendix*, Table S1). The spatial spread was anisotropic, extending further along the medial-lateral axis, consistent with higher horizontal connectivity within each whisker row (38, 39). This anisotropic spread was qualitatively more pronounced at lower frequencies (θ & ß) than higher frequencies (Hγ & uHγ). Thus, like in the rat auditory cortex (17), stimulus-evoked CSEPs recorded by µECoG in S1 are localized to approximately a single functional column and topographically organized, with activity centered on the cortical column corresponding to the deflected whisker.

### Optogenetic Suppression of L2/3 vs. L5 PYR Cells.

**Fig. 2.**
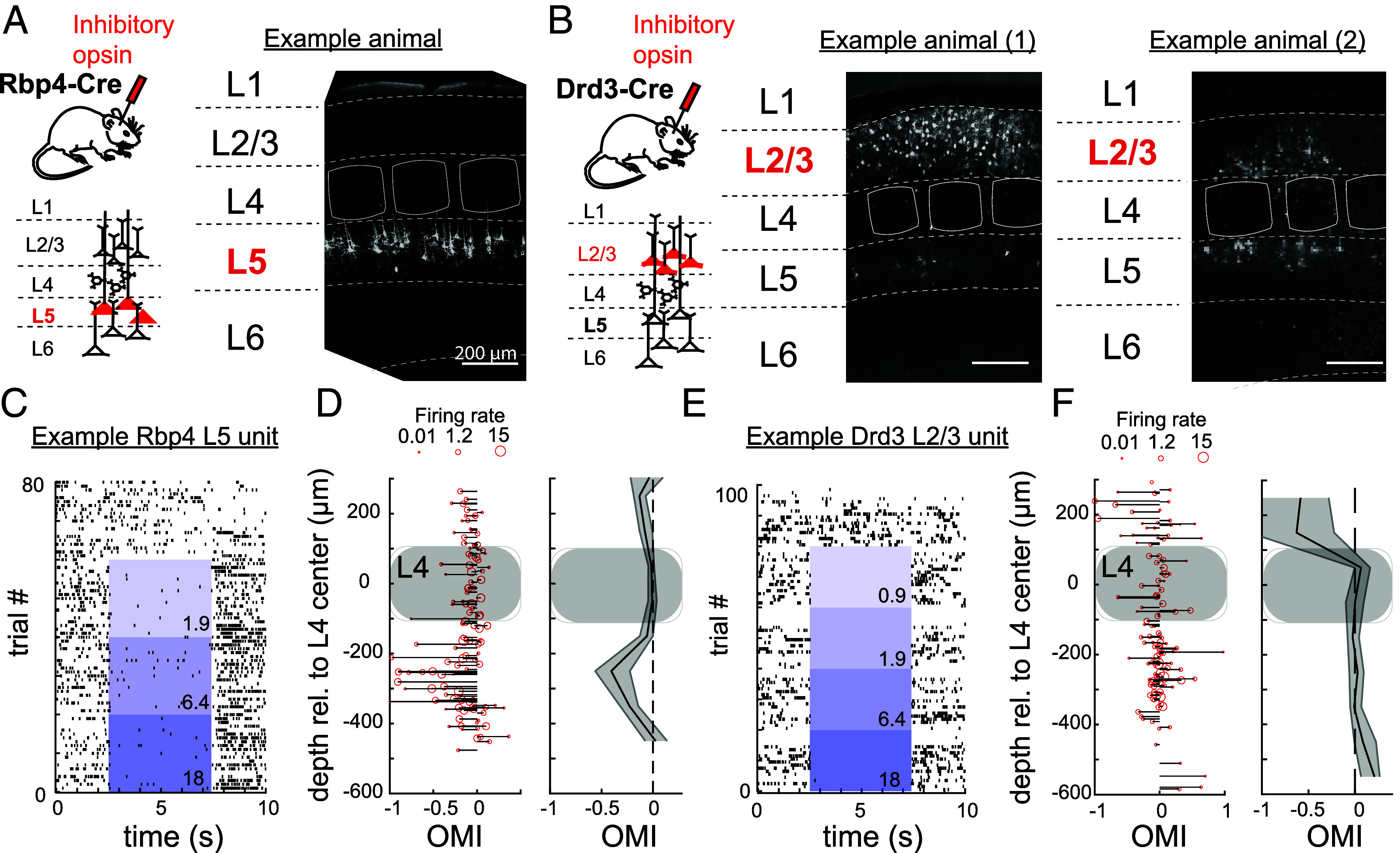
Optogenetic suppression of L2/3 versus L5 PYR cells. (*A* and *B*) Histological sections from three mice, showing endogenous stGtACR2-FusionRed signal. The Rpb4-Cre mouse shows typical L5 expression (*A*), and the two example Drd3-Cre mice (*B*) were chosen to illustrate more selective (1) and less selective (2) expression in L2/3. (*C*) Spike raster from a L5 multiunit displaying optogenetic inhibition in a Rpb4-Cre mouse. Blue shows light at increasing light intensities (darker blues), with numbers indicating light power in mW. (*D*) Population modulation of multiunit activity within Rpb4-Cre mice (five mice and penetrations, 128 units). *Left*, Optogenetic modulation of firing rate (OMI) for each unit across penetrations, as a function of cortical depth. (*Right*) Average across units pooled in 100 µm bins. Shading is 95% confidence interval. (*E*) Same as (*C*) but for a L2/3 multiunit in a Drd3-Cre mouse. (*F*) Same as (*D*) but for L2/3 multiunits in Drd3-Cre mice.

To test the contribution of L2/3 or L5 PYR neurons to whisker-evoked CSEPs, we optogenetically suppressed spiking of L2/3 or L5 PYR cells using the soma-targeted inhibitory opsin stGtACR2. Cre-dependent expression of stGtACR2 was achieved by injection of AAV-hSyn1-SIO-stGtACR2-FusionRed into S1 of Rpb4-Cre mice or Drd3-Cre mice, which target L5 and L2/3 PYR cells, respectively (31, 32). After 14 to 21 d, we performed in vivo neurophysiology experiments. Post hoc histological analysis confirmed dense expression of stGtACR2 in L5 PYR cells in Rpb4-Cre mice, with no expression in other layers (example mouse in Fig. 2*A*, same pattern observed in all mice). In Drd3-Cre mice, L2/3 PYR cells always expressed stGtACR2, but L5 expression was also variably observed in what appeared to be thin-tufted L5 PYR cells in some mice and columns. Thus, some Drd3-Cre mice exhibited selective and dense expression in L2/3 PYR cells (Fig. 2 *B*, *Left*), while others also had expression in L5 PYR cells (Fig. 2 *B*, *Right*).

To test whether stGtACR2 could achieve layer-specific inhibition of L2/3 or L5 PYR cells in Drd3-Cre and Rpb4-Cre mice, we used a 32-channel laminar polytrode (spanning L2/3 to L5) to extracellularly record neuronal units in vivo. Spontaneous and whisker-evoked spiking were monitored, and interleaved trials contained either optogenetic light (at intensities from 0.9, 1.9, 3.5, 6.4, 11.0, or 18.0 mW) or no light (0 mW), applied to the cortical surface centered on the probe insertion site (Fig. 2 *C–F*). After spike sorting (40), we analyzed both single and multiunits and compared spike rates between light- and no-light (control) trials. The laminar location of each unit was determined by depth relative to the center of L4, identified from the pattern of whisker-evoked local field potentials on the probe (*SI Appendix*, Fig. S3). In recordings from Rpb4-Cre mice (n = 5) with histologically confirmed L5 expression of stGtACR2 in the recorded column, we observed individual L5 units whose spiking was suppressed by light (Fig. 2*C* shows one example L5 multiunit in response to multiple light levels). For each unit we calculated an optogenetic modulation index (OMI) that quantified firing rate changes across the full trial duration in optogenetic stimulation trials (all light levels ≥ 6.4 mW) vs. interleaved no-light trials. An OMI of 0 indicates no suppression, while an OMI of −1 indicates complete suppression of spiking by light. Across all 5 Rbp4-Cre mice, L5 units (100 to 400 µm below L4 center) were most strongly suppressed (Fig. 2*D*, OMI = −0.219 ± 0.04, mean ± SEM, n = 56 units, *P* < 0.001 relative to OMI = 0, two-sided nonparametric bootstrap test), while L2/3 units (100 to 400 µm above L4 center) showed a much weaker suppression (OMI = −0.079 ± 0.03, n = 25 units, *P* = 0.016). In contrast, in Drd3-Cre mice (n = 5) that were confirmed histologically to have L2/3 expression but sparse or no L5 expression in the recorded column, L2/3 units were often suppressed by optogenetic light (an example L2/3 multiunit is shown in Fig. 2*E*). Across these Drd3-Cre mice, average spiking was significantly suppressed for L2/3 units (Fig. 2*F*, OMI = −0.615 ± 0.17, mean across n = 18 units, *P* = 0.006), but not for L5 units (OMI = 0.014 ± 0.02 OMI, n = 66 units, *P* = 0.7). The minor suppression of spiking for units in nongenetically targeted lamina may reflect indirect circuit-level effects of target layer suppression. Thus, stGtACR2 suppressed mean firing rate by 22 to 60% in a layer-specific manner: Rpb4-Cre mice showed fairly selective optogenetic suppression of L5 spiking, while Drd3-Cre mice with L2/3-selective expression showed selective suppression of L2/3 spiking.

### Different CSEP Frequency Bands are Modulated by Suppression of L2/3 vs. L5.

**Fig. 3.**
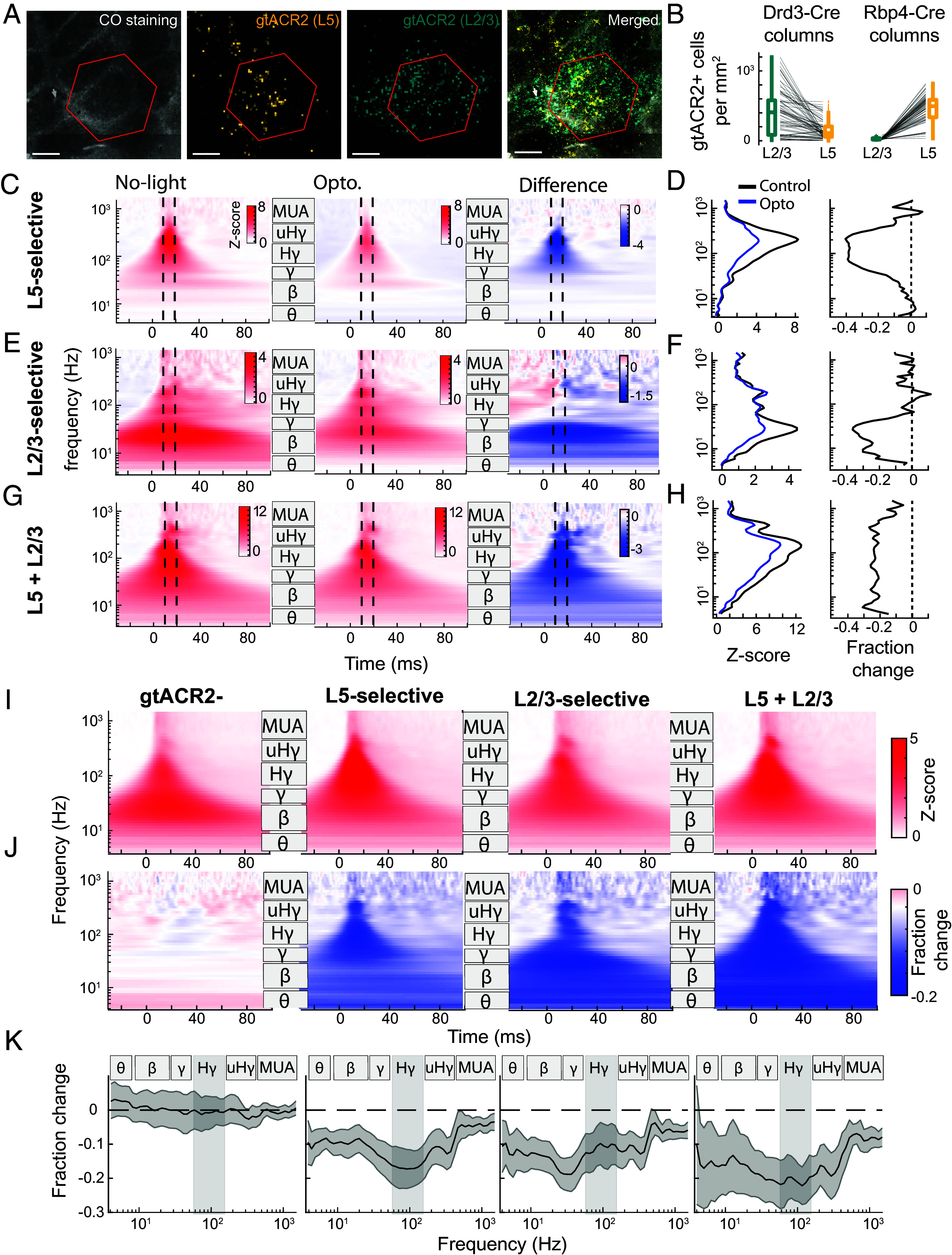
Different CSEP frequency bands are modulated by suppression of L2/3 vs. L5. (*A*) Tangential sections from an example Drd3-cre mouse, with hexagon for quantifying stGtACR2 expression around the D1 peak channel. FusionRed reporting stGtACR2 in L5 (gold) and L2/3 (teal) is shown relative to CO staining in L4 (black). (*B*) Quantification of stGtACR2 in L23 and L5 in all cortical columns from all 17 mice with histological data. (*C*, *D*) example peak channel with selective expression in L5. (*E*, *F*) example peak channel with selective expression in L2/3. example peak channels with expression in both L5 and L2/3 (*G*, *H*). (*C*, *E*, and *G*) Average whisker-evoked CSEP spectrogram for the three example peak-channels, in the no-light condition (“Control”, *Left* column). Light condition (“Opto”, *Middle*) and the difference Opto–Control (“Difference”, *Right*). (*D*, *F*, and *H*) Quantification of CSEP magnitude by frequency for Control and Opto conditions, and the fraction change. These were quantified in the ±5 ms time window around peak response.

To test how layer-specific suppression affects CSEPs, we performed µECoG recordings while applying optogenetic light at varying levels, or no light, through the transparent µECoG array on interleaved trials. Optogenetic illumination covered multiple cortical columns with a diameter of ~0.63 mm (full width at half maximum). These recordings were separate experiments from the intracortical recordings and came from a larger set of mice (10 Rpb4-Cre mice, 12 Drd3-Cre mice, and three uninjected C57BL/6 mice). Experimental variability in the light intensity and viral-mediated opsin expression at each spatial location in S1 will drive variability in optogenetic effects at different peak-channels. We therefore quantified opsin expression in L2/3 and L5 at each peak-channel location in each mouse (Fig. 3 *A* and *B*). To do this, we prepared tangential histological sections (parallel to the pial surface) and measured the density of FusionRed-positive neuronal somata (which marks stGtACR2 expression) in L2/3 and L5. Expression density was quantified in a hexagonal area centered on the cortical column for each peak-channel (e.g., on the anatomical D2 column for the D2 whisker’s peak-channel, Fig. 3*A*). We then classified peak-channels into four groups based on layer-specific opsin expression and light delivery (*Methods* and *SI Appendix*, Table S2):

1) Peak-channels from mice with no stGtACR2 expression (n = 63 channels from three uninjected C57Bl/6 mice, and from 3 Drd3-Cre and 2 Rbp4-Cre mice with failed stGtACR2 expression).
2) Peak-channels with selective expression in L5, but not L2/3 (n = 35 channels from 6 Rbp4-Cre mice).
3) Peak-channels with selective expression in L2/3, but not L5 (n = 25 channels from 6 Drd3-Cre mice).
4) Peak-channels with expression in both L2/3 and L5 (n = 15 channels from 4 Drd3-Cre mice).

We evaluated the impact of optogenetic light in these four groups by analyzing CSEPs evoked by the stimulation of the peak-channel’s whisker. Focusing first on the groups with stGtACR2 expression, Fig. 3 *C–H* shows example peak channels from the groups with either L5-selective, L2/3-selective, or L2/3 + L5 expression. We compared the CSEP spectrogram (z-scored to baseline activity in each frequency) in light-on trials (≥6.4 mW) versus no-light trials by plotting the difference (ΔZ-score) of these spectrograms, and the fractional change relative to no-light trials (using non-Z-scored frequency band power). The ΔZ-score and fractional change were then quantified for all frequencies. In these representative examples, selective L5 suppression led to a reduction in Hγ and uHγ power, selective L2/3 suppression reduced low-frequency activity (θ, β, and γ bands), and combined L2/3 + L5 suppression resulted in broadband attenuation across frequency bands.

We assessed the population average effect of optogenetic suppression on CSEPs within each layer-specific group (Fig. 3 *I–K*). The average CSEP spectrogram across all peak-channels for the no-light condition (z-scored to baseline activity) revealed robust responses to whisker deflection across frequency bands in each group (Fig. 3*I*). Next, for each peak-channel, we computed the fractional change for each frequency relative to no-light trials and the resulting spectrograms were pooled within each group (Fig. 3*J*). We then quantified this fractional change for individual frequencies within a ±5 ms window around the peak of the evoked response (Fig. 3*K*). We found that channels with no stGtACR2 expression had no optogenetic modulation in CSEP power across any frequency band, assessed by the fractional difference in power between light and no-light trials (0.01 ± 1.7% opto-modulation from 4 to 1,500 Hz, mean ± SEM across peak-channels, Fig. 3*K*). Each of the three other groups showed significant differences at all frequencies (95% CI lie below 0 in Fig. 3*K*) with apparently nonuniform suppression across the spectrum (Hγ for L5-selective peak channels, and at lower frequencies for L2/3-selective peak channels).

### L5 vs. L2/3 Preferentially Contribute to High vs. Low-Frequency CSEP Signals.

**Fig. 4.**
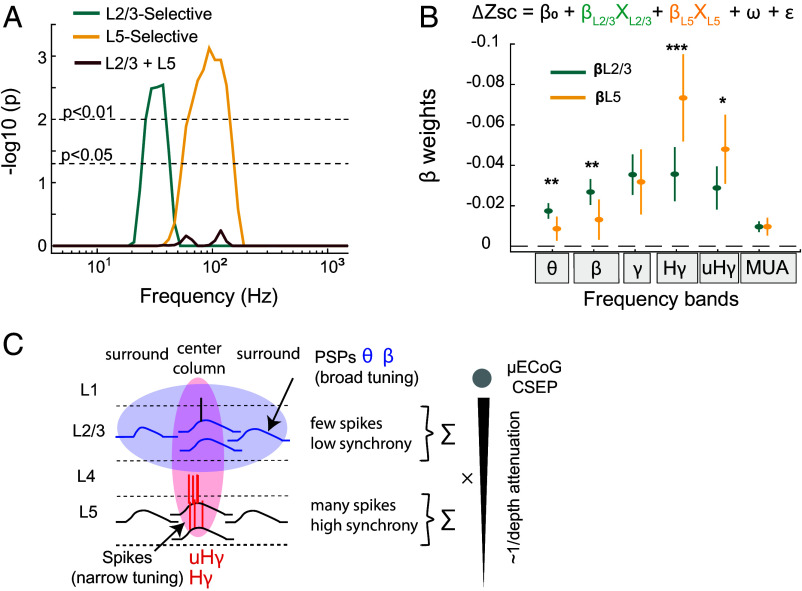
L5 vs. L2/3 preferentially contribute to high vs. low-frequency CSEP signals. (*A*) Statistical significance of frequency-specific suppression within each peak-channel group. Permutation-test results are shown as −log10(*P*-value). These *P*-values quantify whether the observed suppression at a given frequency (fractional change as shown in Fig. 3*K*) is deeper than expected under a null model of uniform suppression across frequencies. Significant frequency-selective suppression was observed for ~25 to 40 Hz for L2/3-selective channels, and for ~60 to 110 Hz for L5-selective channels. (*B*) Mixed effect model of CSEP suppression. We modeled suppression in each canonical frequency band based on putative spiking suppression of L5 and L2/3 PYR cells, estimated from local stGtACR2 expression and light intensity in individual peak-channels. ß weights represent contribution of each layer’s activity to the frequency band in the CSEP. Error bars represent the 95% CI. Statistical tests are conducted using a randomization procedure. (*C*) Conceptual model of possible laminar contributions to ECoG (*Discussion*). (*Left*) Schematic voltage-time traces for individual sources in L2/3 and L5 center and surround columns. L5 PYR exhibits temporally synchronous, high firing rate, columnar tuning and a strong dipole geometry, generating large electrical fields whose Hγ components can be detected at the surface. Local L5 postsynaptic potentials (PSPs) are less concentrated in space (broad tuning). L2/3 PYR exhibit sparser spikes with the contribution to CSEP of the layer being dominated by the broadly tuned PSPs, integrating over longer time windows.

To test if these results reflect a true difference in contribution of L2/3 and L5 to high and low frequency CSEPS, we took two approaches. First, to determine whether frequencies were significantly suppressed within each group, we performed a permutation-based test of whether the suppression observed at a particular frequency is lower than expected from a null model of uniform suppression across frequencies (implemented by shuffling suppression values across frequencies, *Methods*). For channels with selective L5 expression, we observed that the maximum suppression, on average across peak channels, was at 93.8 Hz (17.3 ± 2.9% suppression), and the statistical test showed that the entire frequency band 59 to 148 Hz was significantly suppressed below the null model (*P* < 0.05; one-sided permutation test, Cohen’s d = 1.51 for the entire band, Fig. 4*A*). For channels with selective L2/3 expression, we observed that the maximum suppression was at 37.2 Hz (18.8 ± 2.6% suppression), and the statistical test showed that the entire frequency band 26 to 42 Hz was suppressed below the null model (*P* < 0.05; one-sided permutation test, Cohen’s d = 1.84 for the entire band, Fig. 4*A*). For channels with combined L2/3 and L5 expression we observed a broad-spectrum suppression spanning both low and high CSEP frequencies, with a peak of 22.2 ± 2.8% at 118.1 Hz. However, in this group, no significant local minima were found with the permutation test (all *P* > 0.05). To control for potential confounds associated with differences in evoked response magnitude across genotypes, this analysis was repeated over L5 selective and L2/3 selective groups with matched evoked responses. This analysis showed a similar effect, despite reduced statistical power (*SI Appendix*, Fig. S4). Thus, this demonstrates distinct, frequency-selective suppression of the ECoG CSEP within the L2/3 PYR and the L5 PYR groups of peak-channels.

In a second approach, we explicitly compared the contribution of different layers in a multivariate model. The population-average results above demonstrate that optogenetic suppression of L2/3 and L5 preferentially suppress low and high-frequency CSEPs, respectively. However, there is substantial variability in the level of opsin expression and other factors, which will modulate the relative magnitude of suppression. Therefore, we used a linear mixed-effects model to quantify the relative contribution of L2/3 and L5 to optogenetic suppression across CSEP frequency bands (Fig. 4 and *Methods*). Unlike the population-average approach above, the model accounted for the quantitative level of opsin expression (determined histologically) at each peak-channel location, spatial and trial-by-trial variability in light exposure, and statistical dependence between peak-channels in the same recording session. In addition, the model included the entire dataset with histologically quantified levels of stGtACR2 expression, not just the channels that fit within the four groups defined previously. Optogenetic suppression (ΔZ-score) was modeled using two fixed effects: ß_L2/3_X_L2/3_ and ß_L5_X_L5_ where X_L2/3_ and X_L5_ represent the level of optogenetic inhibitory drive (magnitude of opsin expression in L2/3 or L5 PYR cells times light exposure) at each peak-channel ß_L2/3_ and ß_L5_ are the estimated frequency-specific suppression linked to firing rate reduction in these layers. A nested random effect (whisker identity) controlled for repeated measurements within recordings. The model predictive performance was quantified with leave-one-out cross-validation.

The model was fit independently for each of the six frequency bands (θ, β, γ, Hγ, uHγ, and MUA). When evaluated on held-out data, the models achieved significant predictive performance, with *P*-values <10^−16^ and coefficients of determination (R^2^) ranging from 0.26 to 0.42 across bands (*SI Appendix*, Table S3). In this model formulation, negative ß values represent a suppression of the frequency band in response to light. In all frequency bands, the fitted coefficients ß_L23_ and ß_L5_ were significantly below 0, indicating that suppressing L2/3 or L5 activity both significantly reduced CSEP power in that frequency band (Fig. 4). However, we found a significantly greater contribution of L5 as compared to L2/3 in the Hγ band (ß_L5_/ß_L2/3_ = 2.06; *P* = 0.0007, n = 717 suppression data points from 17 independent recordings, two-sided permutation test corrected for multiple comparisons, see *Methods* and all tests details in *SI Appendix*, Table S3). This was true to a lesser extent for the uHγ band (ß_L5_/ß_L2/3_ =1.67; *P* = 0.018). In contrast, we found a significantly greater contribution of L2/3 as compared to L5 in the θ band (ß_L2/3_/ß_L5_ =2.02; *P* = 0.002) and β band (ß_L2/3_/ß_L5_ = 2.04; *P* = 0.005). Together, these results establish that L5 is the primary source of sensory-evoked Hγ and uHγ ECoG activity (65 to 400 Hz), whereas L2/3 drives lower-frequency components (4 to 30 Hz). This demonstrates a clear laminar dominance in sensory-evoked ECoG activity.

## Discussion

### Resolving the Inverse Problem of CSEPs with Causal Manipulation of Targeted Source Neurons.

Identifying the precise neuronal sources that combine to produce the ECoG CSEP is known as the “inverse problem” for CSEPs. Solving the inverse problem is inherently difficult because diverse combinations of neuronal sources can, in principle, produce similar surface potentials–that is, it is an ill-posed inverse problem (2, 41). Further complications arise from the proximity in time and space of activation of multiple cell types across layers, and the contribution of action potentials, synaptic potentials, dendritic calcium spikes, and other signals within and across cells. Previous efforts to identify the contributions of specific neural layers and sources to CSEPs have primarily relied on correlations between ECoG, intracortical LFP, and spiking in vivo (8, 14, 18) or using computational simulations (17, 42). For example, Leszczyński et al. (14) proposed a prominent contribution from superficial layers to pial broad-band high-frequency activity based on correlational measures (14). While informative, these approaches have inherent limitations. Correlation studies are limited by the near-simultaneous activation of all cortical layers, and fail to systematically account for key biophysical factors like neuronal synchrony, size of produced dipoles (due to neuronal geometry), and total number of neurons (17, 22). Computational models, while offering valuable theoretical insights and predictions, depend on simplifying assumptions that may be inaccurate, such as omitting certain cell types or even whole layers, using biophysically oversimplified neurons, or lacking cross-columnar interactions (17, 42–44). As a result, the origin of Hγ signals (which are widely studied by ECoG) and other frequency bands within CSEPs have remained unresolved.

In particular, it has been unclear whether Hγ arises from very local sources (e.g., a single cortical column) and what is the laminar origin of these signals. To resolve this, we used causal layer-specific manipulation to determine the relative contribution of L2/3 and L5 PYR cells to sensory-evoked CSEPs recorded by µECoG in mouse S1. Our results show that sensory-evoked Hγ CSEP signals are temporally brief and display sharp, column-scale topographical localization (Fig. 1 and *SI Appendix*, Fig. S1). Using layer-specific optogenetic suppression of L2/3 or L5 PYR cells (Fig. 2), we found that L5 suppression preferentially decreased the high frequencies (Hγ and uHγ bands) within CSEPs, while L2/3 suppression preferentially decreased lower frequencies (θ and β) (Figs. 3 and 4). Experimentally, the soma-targeted stGtACR2 activates a chloride conductance that is preferentially localized at the soma and proximal dendrites, hyperpolarizing the membrane potential and shunting incoming synaptic current and thereby reducing firing rate. Using a mixed effects model, we found that L5 exerts twice the influence (i.e., beta coefficient), relative to L2/3, on Hγ-uHγ CSEP bands, while L2/3 contributes twice as strongly to the θ-β bands (Fig. 4*A*). Thus, L2/3 and L5 PYR cells differentially generate low- and high-frequency CSEP components (Figs. 3 and 4). These results experimentally validate the findings of a prior computational study that L5 PYR neurons in the local cortical column are the primary contributors to high-frequency CSEP activity (17), and do not support alternative hypotheses that all layers contribute equally, or that L2/3 is the predominant source of high-frequency CSEPs (8, 14, 42–44). These differences from prior correlative studies exemplify the need for causal experiments, such as the optogenetics approach used here, combined with detailed biophysical modeling.

Our finding that L5 dominates high-frequency ECoG signals may be a consequence of the well-established neocortical principle that while both L2/3 and L5 PYR cells exhibit PSPs to feedforward (sensory-evoked) columnar input, L2/3 PYR neurons exhibit fewer spikes and a sparser sensory code than L5 PYR neurons (45), while L5 PYR cells produce more total spikes and a higher mean firing rate per neuron, including spike bursts (46). These differences in spiking statistics are conserved across cortical areas and species (14, 45, 47–49), and will likely have important consequences for extracellular field generation. Specifically, we propose that temporally dense spiking from L5 results in more synchrony of population firing rates and thus generates large electrical fields dominated by high-frequency power. In contrast, temporally sparse spiking from L2/3 results in less synchrony of population firing rates and thus generates electrical fields dominated by low-frequency power because of PSPs whose slow kinetics allow them to be integrated over extended time windows (2, 24). Our findings map directly onto this canonical principle (Fig. 4*C*). Suppressing L2/3 PYR neuron activity primarily attenuated low-frequency components (4 to 30 Hz) of the sensory-evoked ECoG signal, which were spatially elongated along the whisker row (Fig. 1*H*), consistent with an origin in PSPs driven by anisotropic connectivity of L2/3 cross-columnar projections (38, 39). In contrast, suppressing L5 PYR neuron activity primarily attenuated high-frequency components (65 to 450 Hz), which were sharply localized in space, consistent with synchronous firing, strong dipoles, and narrow sensory tuning of firing rates in L5 neurons. This conceptual model may therefore explain how L2/3 could contribute predominantly to low-frequency CSEPs via broadly tuned PSPs (50–52), while L5 contributes to high-frequency signals via narrowly tuned synchronous spiking (46, 53).

### High-Gamma Activity Preferentially Reports Cortical Column Output.

Together, our findings indicate that Hγ activity within sensory-evoked CSEPs does not reflect the simple average activity across layers, but instead more strongly reflects L5 PYR spiking activity compared to other sources. In the sensory cortex, the canonical feedforward cortical microcircuit starts in L4, the main thalamic recipient layer, and progresses to L2/3 and then L5 (54, 55), though actual processing is more complex (56). As information propagates through this laminar hierarchy, L2/3 and L5 perform nonlinear integration of feedforward input with contextual information from nearby columns and additional context and expectations from top–down inputs (57–59). The results of these columnar computations are then broadcast to the rest of the brain, shaping behavior and sensory decisions (60–62). L2/3 cells represent an intermediate stage of intracolumnar processing and provide output to higher cortical areas, while L5 cells are the final output to cortical and subcortical targets, including higher-order thalamus, striatum, and motor-related structures in the midbrain and brainstem (63) and are most related to perceptual decisions and motor control (60).

In this standard model of sensory-evoked columnar processing, ECoG Hγ provides a biomarker for the final L5 output of columnar computation. In contrast, lower frequency bands may reflect more intermediate stages of computation, e.g., in L2/3, and from input PSPs prior to cellular processing into spike trains. Importantly, we found sensory-evoked Hγ in S1 is transient and nonoscillatory, and is likely to reflect brief periods of feedforward-driven, synchronous high firing in L5 PYRs of individual columns. This is distinct from oscillations, which may emerge from feedback interactions and may contribute to interareal communication, and which are often inferred from spectral structure in the ECoG and LFP literature (2, 64, 65). While our analysis decomposes CSEPs into canonical frequency bands, we did not observe oscillations per se, but transient increases in power at those frequencies, consistent with transient activity bursts that are known to transmit information in the sensory cortex, which have been proposed to increase efficiency of information transmission to downstream targets (66–68).

Hγ activity in CSEPs is associated with a variety of perceptual, behavioral, and cognitive processes in animals (21, 69, 70) and humans (7, 10, 13, 71). Our results and prior simulation indicate that depth is only a minor factor in determining the contribution of a layer to CSEPs compared to the number of cells and their synchrony. This scaling principle should generalize to other time-locked evoked ECoG responses, in humans and other animals, because of analogies in cortical organization and coding across species. Indeed, the temporally punctate and spatially localized evoked responses we observe in mouse S1 are qualitatively similar to tone responses in human A1 (72), as well as high-gamma activity in the human speech motor cortex during consonant-vowel production (13). As such, our results are likely to generalize to evoked activity in human contexts under analogous conditions. The finding that Hγ is a biomarker of columnar output, rather than internal columnar processing, also provides an explanation for the remarkable tuning of Hγ to sensory and motor variables. In sensory systems, this includes sharp sensory tuning of Hγ in animal models (17, 26), which tracks the sharper sensory tuning of L5 neurons than L2/3 neurons (35, 73, 74), and sharp tuning for speech acoustics in humans (75). In motor areas, such as those controlling vocal tract articulators in humans (13), sharp Hγ tuning may reflect the motor output of L5 neurons rather than preparatory activity, which may explain the success of decoding motor commands from these ECoG signals. The paradoxical observation that ECoG Hγ has sharp, topographically organized tuning may thus reflect its readout of finely tuned activity and precise topographical projections of L5 neurons.

### Open Questions and Future Directions.

Together, our findings indicate that sensory-evoked Hγ ECoG activity is most parsimoniously interpreted as a readout of L5 columnar spiking output rather than as a marker of input drive or internal processing. Our experimental design investigated single-column feedforward processing, and does not make predictions for intercolumnar contributions to CSEP frequency bands, including feedback processing, that have been discussed elsewhere (14). The relative contributions of within column vs. across column PSPs in L2/3 that drive low-frequency ECoG signals remain unclear, and more detailed modeling and higher-resolution manipulations will be essential to disentangle these sources. In the MUA (>500 Hz) range of the CSEP that likely reflects overlapping spike waveforms, we observed only modest effects of L2/3 or L5 suppression. Looking ahead, how broadly do these laminar–spectral relationships generalize across cortical regions and behavioral states? Although laminar organization is conserved throughout the neocortex, regional differences in layer thickness and cellular composition may shape how different lamina are reflected in frequency bands of CSEPs, and hence how those frequency bands map onto cortical processing.

## Conclusion

Our findings offer a functional interpretation of distinct ECoG frequency bands as biomarkers of neural activity in different cortical layers. ECoG Hγ preferentially reflects L5 columnar output, while low frequency signals are more linked to L2/3, and hence may reflect intracolumnar processing. Thus, different frequency bands of CSEPs may be biomarkers of distinct computational stages of columnar processing. Enhancing our understanding of the sources of ECoG signals (76), especially from high-resolution, large-scale recording devices (77, 78), could enhance performance of neuroprosthetic systems (79) and diagnostic tools. Large scale µECoG systems that densely tile multiple cortical areas may be a valuable research tool by enabling millisecond resolution columnar-scale readout of different stages of laminar processing across cortical regions.

## Methods

### Animals.

Data from three wild type (WT), 18 Rbp4-Cre, 17 Drd3-Cre animals were used in this study. All animal procedures were performed in accordance with local regulations as described in the IACUC Protocol # AUP-2016-02-8351-2. (Institutional Animal Care and Use Committee). WT and Drd3 [(Tg(Drd3-cre) KI196Gsat/Gene Expression in the Nervous System Atlas (GENSAT) project through the Mutant Mouse Regional Resource Centers (MMRRC) (strain number 034610) JAX #)] (Gensat MMRRC, strain number 034610) mice were C57Bl6 background while Rbp4 [(Tg(Rbp4-cre) KL100Gsat/(GENSAT line # KL100)] were on Agouti/C57Bl6 mixed background. Mice were 2-11 month old and were either male or female.

### Surgeries.

An initial viral injection was performed in Rbp4-Cre and Drd3-Cre mice. Mice were anesthetized with isoflurane (induction at 5%, maintenance at 2%) and placed in a stereotaxic frame over a feedback-controlled heating pad set to 37 °C. Eyes were protected from desiccation using a petroleum-based eye ointment. General analgesia was provided with Meloxicam (5 mg/kg, subcutaneous), buprenorphine (0.1 mg/kg, subcutaneous), and dexamethasone (4 mg/kg, subcutaneous). The incision area was shaved and disinfected with betadine, and lidocaine was injected along the midline under the scalp (1%, 0.1 mL, subcutaneous). A 10 mm incision was made to expose the skull. A burr hole was drilled at the coordinates of C1 or C2 whisker barrel in the left hemisphere (AP 1.4, ML −3.3 relative to bregma) using a dental drill. A beveled glass pipette with a diameter below 30 µm was inserted at a depth of 400 to 500 µm. Between 400 to 600 nL of the viral construct pAAV_hSyn1-SIO-stGtACR2-FusionRed (a gift from Ofer Yizhar; Addgene viral prep # 105677; RRID:Addgene_105677) was injected at a rate of approximately 60 nL per min. The pipette was removed 3 min after the injection concluded. The skin was sutured, and the animal was placed in a recovery cage, half over a heating pad, until fully awake. Animals were monitored for 3 d postsurgery, with meloxicam (5 mg/kg, subcutaneous) administered once daily. Optimal transfection density and stability were achieved by diluting the viral construct in Ringer’s solution to a final concentration of 1 × 10^12^ vg/mL, and waiting 14 to 21 d postinfection before recording. Control animals received either sham viral injections (Drd3-Cre and Rbp4-Cre mice) or no injection at all WT.

Following the incubation period, The mouse was prepared using the same protocol as for viral injection until incision. Then the skull was cleaned using a scalpel, the skin was secured away from the skull using surgical glue, and the bone was maximally thinned on the edges of a rectangular craniotomy using the dental drill. The rectangular craniotomy area measured approximately 5 mm (Medio-lateral axis) by 3 mm (Antero-posterior axis) and was centered to a point 1 mm lateral to the C1 whisker barrel. A burr hole was drilled in the frontal region of the ipsilateral hemisphere to insert a 30 gauge silver-wire connected to a golden pin which would serve as reference for the electrophysiological recordings. Dental cement was used to seal a custom metal headpost in the back of the skull. The area was kept moistened with saline for at least 5 min to limit bleeding at bone removal. Bone flap was removed with a tweezer and the animal placed in the setup for electrophysiological recording.

### Electrophysiological Recordings.

The protocol for simultaneous electrophysiological recording and optogenetic inhibition has been described in detail previously (34). Briefly, during the recording session, animals were maintained in a lightly anesthetized state (Isoflurane 0.5 to 1% in 0.4 L/min O_2_), and placed on a feedback-controlled heating pad set to 37 °C with head-fixation. Chlorprothixene was administered (1 mg/kg, intraperitoneal) to help maintain anesthesia at a lower isoflurane level.

Neural data from ECoG were acquired using a custom-designed 128-channel µECoG grid (E128-200-8-40-HZ64×2, Neuronexus, Ann Arbor, MI, United States) connected to a 128-channel headstage (SpikeGadgets, San Francisco, CA, United States). The grid was placed on the moistened brain and carefully moved in a medio-lateral direction. Once in position, the remaining saline solution was aspirated, and the grid was gently pressed against the brain in a latero-medial movement, allowing it to bend slightly to better conform to the shape of the mouse brain, ensuring optimal contact between electrodes and the cortical surface. No additional saline was added during the rest of the ECoG recording. If needed, the grid was repositioned following a short somatotopic mapping session (20 to 30 deflections per whisker) to confirm proper positioning. Electrode contacts on the grid had an exposed diameter of 40 µm with a 200 µm interelectrode pitch. Data were recorded at a sampling rate of 30 kHz using Trodes software version 2.3.3 (SpikeGadgets, San Francisco, CA, United States), downsampled to 3 kHz, and saved in Neurodata Without Borders format.

Neural data from the laminar probe recording were collected either in separate animals or after the ECoG recording session. We used 32-channel probes (A1×32-Poly2, Neuronexus, Ann Arbor, MI, United States) and a 32-channel headstage (SpikeGadgets, San Francisco, CA, United States). Duratomy was performed locally with a 30G hypodermic needle, and the probe was inserted at a speed of <2 µm/s. The electrodes were arranged on the probe in a staggered layout every 25 µm over a total length of 800 µm, enabling simultaneous sampling of supragranular, granular, and infragranular layers. Recording began 10 min after the probe was fully inserted. Shielding and grounding of the setup minimized significant 60 Hz noise in the electrophysiological recordings.

Once the recording devices were in place, optogenetic light was directed at the cortical surface, and its position adjusted to match the expression area. Light from a 473 nm LED was collected in a 1 mm diameter optical fiber using a tandem of aspheric lenses. Light from the fiber was delivered through a lens and focused on the surface to create a ~0.63 mm diameter disk (FWHM). In optogenetic trials, light was applied for 4.9 s, starting 100 ms before the first whisker stimulus and ending 450 ms after the last whisker deflection in the trial. Optogenetic light intensities were randomly varied from trial to trial at one of the following values: 0 (control), 0.9, 1.9, 3.5, 6.4, 11.0, or 18 mW. In 25% of trials, light was applied without whisker stimulation.

An array of 3 × 3 whiskers was inserted into glass capillaries attached to individually controlled piezo actuators. Whisker deflections were designed as 12 ms back-and-forth pulse deflections. A 10th line was used as a blank stimulus, randomly intermingled with whisker pulses. Within each trial, 12 whisker pulses were randomly intermingled and delivered with a 350 ms interpulse interval over the total 4.9 s duration of the trials. Trials were separated by a 5 to 7 s intertrial interval.Tactile stimuli were created through custom Igor code and delivered to the piezo actuator at a sampling frequency of 200 kHz. Onset times of whisker stimulation were recorded as Transistor-Transistor Logic pulses in the electrophysiological system with 1 ms precision, allowing for later alignment of stimulus-evoked CSEPs. The intertrial interval was varied between 5 and 8 s. On average, 289.3 ± 11.4 trials (mean ± SEM) were recorded per animal.

### Analysis Code.

All analyses were performed using custom code in MATLAB.

### Histology.

In a subset of 17 animals, 3 to 5 distinct points in the brain were marked with DiO to facilitate later alignment of histological slices. Animals were euthanized by isoflurane inhalation at the end of the recording session, following the ACUC guidelines. The brain was collected and immersed in 4% Paraformaldehyde. The next day, neocortical tissue around the craniotomy area (~6 × 4 mm) was extracted, flattened between two microscope slides, and placed in a 30% sucrose solution for at least 48 h. The brain was then frozen and sliced tangentially to the surface in 50 µm sections. Slices were stained with cytochrome oxidase (Cytochrome C from equine heart; Medix Biochemica, Finland) to reveal the position and contours of the whisker barrels in L4 slices.

Images of each slice were taken using a confocal microscope and realigned along the XY dimension with the EMTrak2 plugin (80) via DiO labeling and blood vessels. Slices above the barrel labeling are considered to belong to L2/3, while slices below the barrels are categorized as L5. In each slice, stGtACR2+ neurons were detected using custom MATLAB software, which identifies them based on shape, size, and contrast to the background. Detection in each slice was verified, and if necessary, the detection parameters were manually adjusted to ensure high-quality results. For each cortical column, we counted the stGtACR2+ neurons within a circular area with a radius equal to the distance between two barrels, thereby encompassing the cortical column and its immediate surrounding region. For each cortical compartment (supragranular and infragranular layers), we measured the maximum density of stGtACR2+ neurons across slices within that compartment. This histological analysis provides a single stGtACR2 expression density value for each cortical compartment within each cortical column.

### Analysis of Spiking Data.

Spike sorting was carried out using Kilosort 3, following the standard preprocessing and analysis described in the reference article (40). All recordings were processed identically using the same thresholds and detection settings. Drift correction was disabled, as it introduced artifacts in recordings with sparse spiking activity. All units assigned by Kilosort3, including unsorted ones, were included in the optogenetic modulation analysis. To avoid contamination by noise, a minimum spike amplitude threshold of 15 arbitrary units was applied to exclude false detections. To identify the center of L4, each electrode voltage time series was Z-scored, and artifacts (voltage changes exceeding 20 SD) were removed. The depth corresponding to the peak power in the 65 to 300 Hz range was identified and used as the L4 center. This frequency peak coincided with a current sink in the Current Source Density profile, as illustrated in *SI Appendix*, Fig. S3. The OMI was calculated as OMI = (FR_light-on_ + FR_no-light_)/(FR_light-on_ − FR_no-light_) where FR indicates the average firing rate across trials.

### Preprocessing and Spectral Analysis.

Preprocessing of the ECoG data was performed as previously described (17). In brief, for each channel, we applied a common average reference subtraction on the 3 kHz signal, subtracting the median of 128 channels. Then a spectral decomposition was performed using a wavelet morse decomposition. Spectral decomposition was performed between 1 Hz and 1.5 kHz with six frequency bins per octave. We considered only the real part of the wavelet decomposition. Each frequency band was individually Z-scored based on the statistics of the baseline activity. For baseline statistics we considered all epochs of time with neither light nor whisker stimuli within the previous or following 1 s. We computed standard deviations and average from this baseline activity in a rolling 60 s long window, and separately for each wavelet frequency. Z-scoring removes the characteristic power law P ~ 1/f^a^ fall-off found in brain electrical fields and highlights sensory-evoked change in the frequency components. The apparent prestimulus “smearing” of evoked activity in low-frequency bands is due to the larger time windows and bandwidths characteristic of these frequencies. Finally, for calculation of a full frequency band (e.g., Theta), we averaged the activity over all Z-score whose center frequency falls within the frequency band. We used the following frequency bands: θ (4 to 8 Hz), β (10 to 27 Hz), γ (30 to 57 Hz), Hγ (65 to 170 Hz), uHγ (180 to 450 Hz), and MUA (500 to 1,500 Hz). Response to stimuli were measured as ±5 ms around response peak time. Response peak time was computed as maximum Z-score in the high-gamma activity band in the 50 ms following the stimulus onset. Data from both single-units and multiunit activity were included in the analysis of laminar probe recordings.

### Analysis of Whisker Tuning and Channel Selection.

One channel was selected to represent each whisker, termed the whisker’s peak-channel. The peak-channel was defined by the user as a manual click at the center of the functional responses observed in the combined Hγ and uHγ bands over the S1 area. Manual selection allowed to avoid unresponsive or broken channels and generated channel selection most coherent with the somatotopic map, as compared to other means of calculation (*SI Appendix*, Fig. S1). For further analysis, a criterion of responsiveness (average Z-score across all frequencies bands >1) was applied to include only strongly whisker responsive peak-channels. It included 271 out of 324 peak-channels.

### Analysis of Optogenetic Effect.

Optogenetic effect magnitude was quantified either as a difference in the Z-score, or as a ratio. Given that Z-score can approximate null values, it cannot reliably be used to compute ratios. Instead the ratio of suppression was calculated from the wavelet raw power, separately for each frequency band.

Grouping of the peak-channels for population analysis (Fig. 3) was made using the same reasoning as for the statistical model. Inclusion in the group requires putative suppression in the defined layers. The suppression of firing rate in a layer depends on the necessary interaction between the opsin expression and the presence of optogenetic light. We used the following criteria of optogenetic light exposure and laminar stGtACR2 expression to constitute the four groups: Control group: no expression of stGtACR2 in the whole animal. L2/3 suppression group: criterion 1) (stGtACR2 expression in L2/3 x λ_spatial_) is above the 70 percentile across all peak-channels, and criterion 2) not in the L23 & L5 suppression group below. L5 suppression group: criterion (stGtACR2 expression in L5 × light level) is above the 70 percentile across all peak-channels. L2/3 & L5 suppression group: criterion stGtACR2 expression in L5 × stGtACR2 expression in L2/3 × light level is above the 90 percentile across all peak-channels, nonoverlapping with the other groups.

To test if suppression was frequency specific, we performed a permutation-based test of global minima on the suppression curves (fractional change: opto/no-opto). The null hypothesis is that suppression is uniformly distributed across frequencies. For each expression group, as defined in Fig. 3, we generated null distributions by repeating 20,000 times the following procedure: 1) shuffle frequency labels for each peak-channel of the group, 2) compute the average spectrum of suppression (across peak-channels) and 3) determined the global minimum i.e., suppression level at the most suppressed frequency. This method provides the null distribution of expected global minimum if suppression was homogeneous across frequencies. We then compared the experimentally observed suppression at each frequency to this null distribution. This provided a one-sided estimate of whether an experimentally observed suppression is significantly deeper than expected from the null hypothesis, relative to the full spectral profile.

For the analysis in Fig. 4*B*, we used a mixed effect model to assess the contribution of each layer to optogenetic modulation of a peak-channel’s average CSEP (81). The dependent variable, ΔZsc, is the difference in sensory-evoked activity between light and no-light conditions for a given peak-channel at a given optogenetic light intensity (n = 717 suppression data points from 153 peak-channels in 17 recordings, multiple intensities per peak-channel). For a given peak-channel, ΔZsc is fitted from the putative suppression of AP activity in the different layers as

[1] $$\Delta \mathrm{Zsc}=\beta_{0}+\beta_{L2/3}X_{L2/3}+\beta_{L5}X_{L5}+\omega +\varepsilon .$$

Here:

• ω represents a nested random effect (whisker identity in the recording).
• ε is the residual error term.
• β_L2/3_ and β_L5_ are the model parameters (i.e., weights) estimating the contribution of each layer to ΔZsc.
• X_L23_ (in L2/3) and X_L5_ (in L5) represent the putative suppression of firing rate in PYR neurons in each layer. The putative suppression of PYR neurons depends on the interaction between the exposition to optogenetic light at the column underneath (λ_L2/3_ and λ_L5_) and the laminar opsin expression in L2/3 (stGtACR2_L23_) or in L5 (stGtACR2_L5_).
X_L23_ (in L2/3) and X_L5_ (in L5) represent the putative suppression of firing rate in PYR neurons in each layer. The putative suppression of PYR neurons depends on the interaction between the exposition to optogenetic light at the column underneath (λ_L2/3_ and λ_L5_) and the laminar opsin expression in L2/3 (stGtACR2_L23_) or in L5 (stGtACR2_L5_).

[2] $$\begin{array}{c} X_{L23}=\lambda_{L2/3}\times \mathrm{stGtACR}2_{L23}\\ X_{L5}=\lambda_{L5}\times \mathrm{stGtACR}2_{L5} \end{array}.$$

Where:

• stGtACR2_L23_ and stGtACR2_L5_ are estimated as the densities of stGtACR2-expressing neurons per mm2 in L2/3 and L5 respectively (see *Histology* section for details).
• λ_L2/3_ and λ_L5_ are unit-free numbers estimating the light reaching neurons in L2/3 and L5, computed as

[3] $$\begin{array}{c} \lambda_{L2/3}=\lambda_{\mathrm{spatial}}\times \lambda_{\mathrm{condition}}\times F_{L23}\\ \lambda_{L5}=\lambda_{\mathrm{spatial}}\times \lambda_{\mathrm{condition}}\times F_{L5} \end{array}.$$

λ_spatial_ is measured as the artifact produced by light in the peak-channel. As light was delivered as a square pulse, it created a sizable artifact at light onset. λ_spatial_ was computed as the Pearson’s R between the optogenetic pulse first derivative and the raw voltage first derivative yielding an area of light clearly localized and matching the position of the optical fiber. λ_condition_ is the light magnitude delivered on the condition (0, 0.9, 1.9, 3.5, 6.4, 11.0, or 18 mW). F_L23_ and F_L5_ are the irradiance fraction of the light reaching the mean depth of the layer (F_L23_ = 0.39 at 200 µm for L23 and F_L5_ = 0.19 at 550 µm for L5) as predicted by the Stanford scale (https://web.stanford.edu/group/dlab/cgi-bin/graph/chart.php). Finally, as we observed saturation in the reduction of neuronal firing rate as a function of light intensity, we applied a ceiling to both λ_L2/3_ and λ_L5_ at a value of 0.0257 (arb. units), corresponding to 3.5 mW of light intensity in the spatially most illuminated channel. This is the level above which we observe no increase in suppression with increasing light intensity. To compute the proportion of variance explained, we used a leave-one-out procedure and calculated the coefficient of determination R^2^ = 1 − RSS/TSS with RSS is the residual sum of squared error and TSS the total sum of squared error. To estimate statistical significance of the difference between β_L2/3_ and β_L5_, we created a null distribution by shuffling randomly the peak-channels order in the matrices X_L2/3_ and X_L5_ and refitting the model 10^5^ times. The experimentally observed difference was compared to the null distribution, and a statistical p-value computed as *P* =2 × (1− |percentile|), to correspond to a two sided test.

## Supplementary Material

Appendix 01 (PDF)

## Data Availability

Electrophysiology recordings are deposited in FigShare https://doi.org/10.6084/m9.figshare.31684585 (82).
