# Supplementary material for: Distinct laminar origins of sensory-evoked high-gamma and low-frequency ECoG signals revealed by optogenetics
Source: Proc Natl Acad Sci U S A. 2026 Apr 1;123(14):e2516293123. doi: 10.1073/pnas.2516293123 (PMC13056151; doi:10.1073/pnas.2516293123)
Supplement: Supplementary file 1 — Appendix 01 (PDF) [file pnas.2516293123.sapp.pdf]

Supplementary information

Distinct laminar origins of high-gamma and low-frequency ECoG signals revealed by optogenetics

Pierre-Marie Garderes[1], Daniel E. Feldman[1,2*], Kristofer E. Bouchard[2,3,4,5*].

Supplementary Table 1. Friedman's post-hoc comparison statistics of spatial spread

Frequency group 1	Frequency group 2	Lower limit	grp1 - grp2 (rank)	Upper limit	p-value
θ	Hγ	0.85	1.31	1.76	<0.001
θ	uHγ	0.64	1.09	1.55	<0.001
β	Hγ	0.68	1.14	1.59	<0.001
β	uHγ	0.46	0.92	1.38	<0.001
γ	Hγ	-0.03	0.42	0.87	0.09
γ	uHγ	-0.25	0.20	0.66	0.79
MUA	Hγ	0.72	1.17	1.63	<0.001
MUA	uHγ	0.50	0.96	1.42	<0.001

Supplementary Table 2. Group by layer expression.

Group	Number of peak-channels	Animals breed	Criteria (Histologically confirmed)
ACR2 - Control	n = 63	N = 3 WT, 3Drd3, 2 Rbp4	no expression
ACR2+ L2/3	n = 25	N = 6 Drd3	>70th percentiles L2/3, and not in ACR2+ L2/3 x L5 group
ACR2+ L5	n = 35	N = 6 Rbp4	>70th percentiles L5
ACR2+ L2/3 x L5	N = 15	N = 4 Drd3	>90th percentile L5 x L2/3

Supplementary Table 3. Statistical details of ßweigths comparisons

Metric	*Theta*	*Beta*	*gamma*	*Hγ*	*uHγ*	*MUA*
Ratio BL5/BL23	*0.50*	*0.49*	*0.90*	*2.06*	*1.66*	*1.00*
Percentile BL23 -BL5	*99.95*	*99.86*	*68.57*	*00.01*	*00.64*	*48.65*
Two-sided p-value	*0.0010*	*0.0028*	*0.6286*	*0.0002*	*0.0128*	*0.9730*
Adjusted p-values (Benjamini-Hochberg)	*** 0.0023*	*** 0.0049*	*0.7334*	**** 0.0007*	** 0.0179*	*0.9730*
Coefficient of determination R^2 (held out)	*0.43*	*0.35*	*0.26*	*0.34*	*0.28*	*0.27*

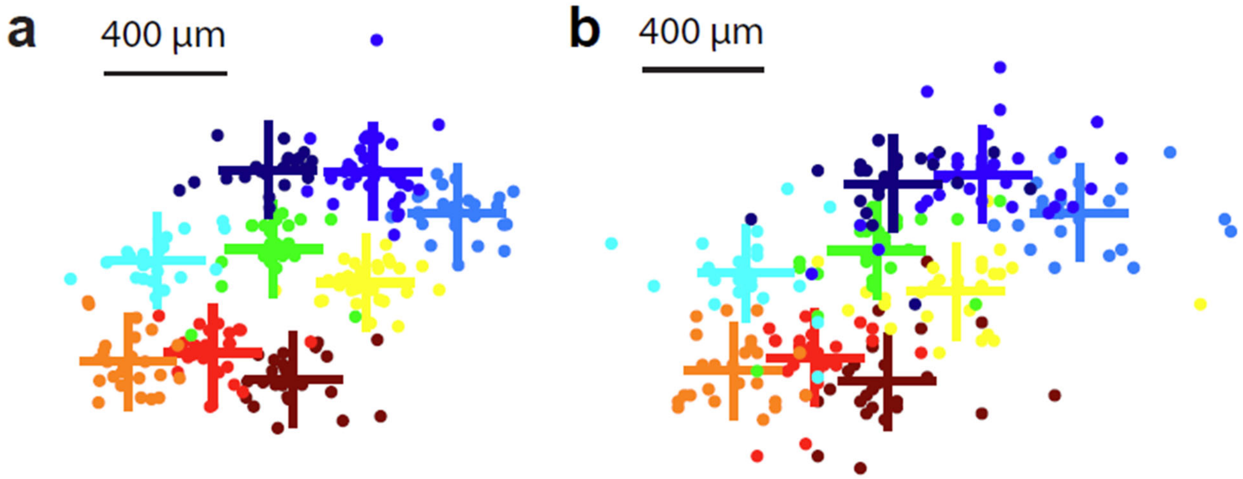

Supplementary Figure 1: Peak Channel selection

Manual and automated methods for peak-channel selection both show clear somatotopy. **a**, Manual selection which was used for all analyses using peak-channels throughout the study. **b**, Spatial peak of Hγ activity also displays robust somatotopy, with individual points more scattered than in the manual method. This difference may arise from electrode properties or varying contact with the underlying tissue. Both maps were aligned across animals by recursively minimizing the Euclidean distance between all whiskers and their averaged position in the pattern, with a resolution of 20 µm (interpolated at 10x).

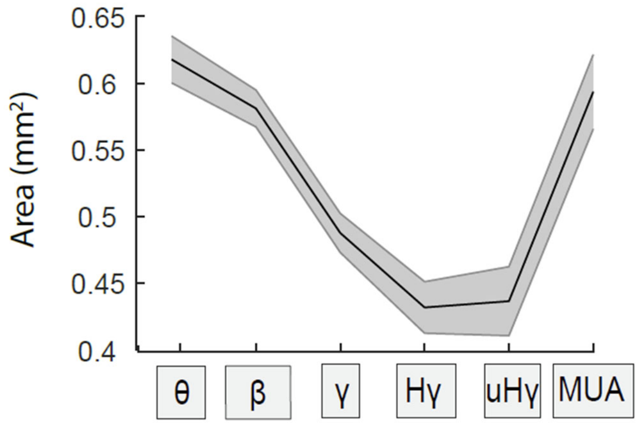

Supplementary Figure 2: Spatial spread of stimulus-evoked activity across different frequency bands in CSEPs.

Spread was quantified as the area where responses exceeded half the peak activity in the endogenous frequency, averaged across responsive peak-channels. Error shades represent sem across n = 271 peak-channels. This pattern is qualitatively similar to observations in the rat auditory cortex. The rise in MUA may, in part, reflect a reduced signal magnitude in that frequency range.

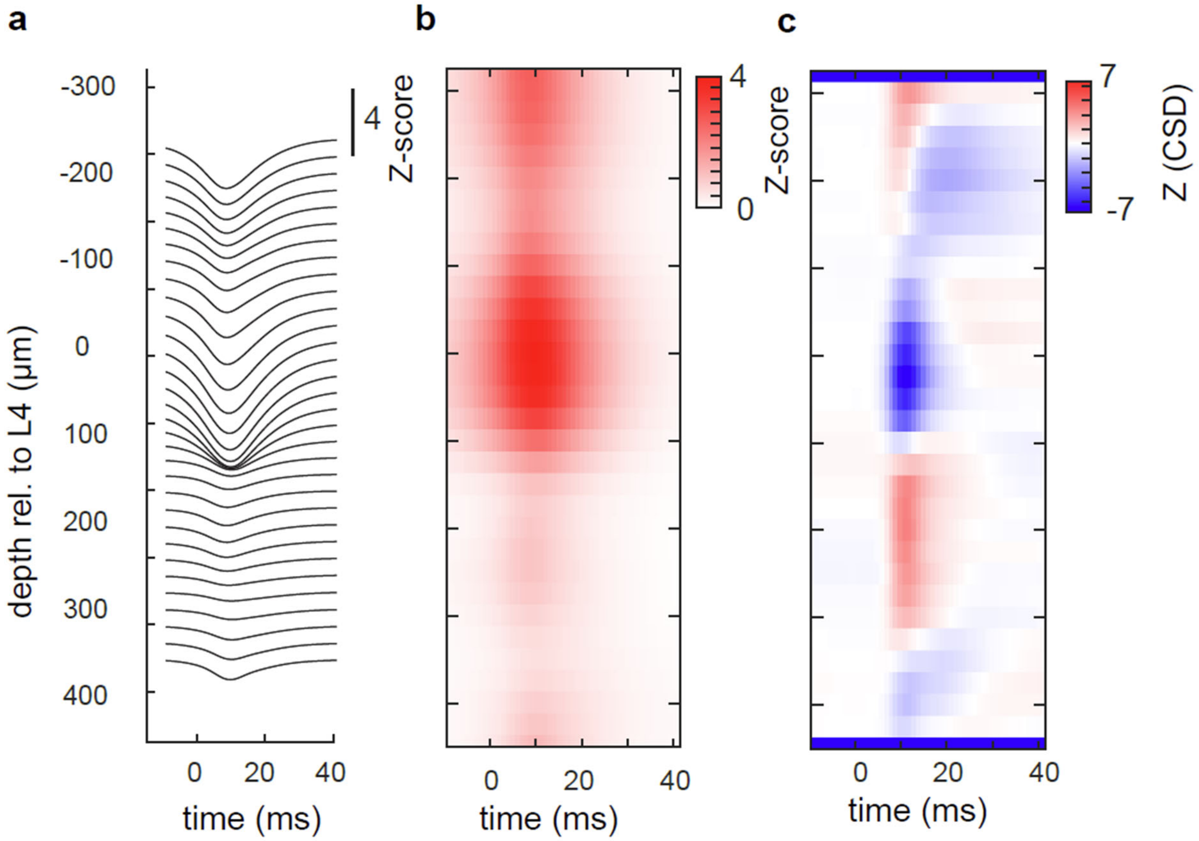

Supplementary Figure 3. Localization of layer 4 center from laminar recordings.

a, Peri-stimulus voltage traces from 32 channels in an example electrode penetration, averaged across trials for the preferred whisker. Signals were Z-scored and bandpass filtered between 1–300 Hz. **b**, Corresponding high-frequency activity used to localize the center of layer 4. The depth with maximal energy in the 10–20 ms window post-stimulation was identified as the L4 center. **c**, Corresponding current source density (CSD) analysis, computed as the second spatial derivative of the voltage signal. Traces were filtered (1–300 Hz) and smoothed in both spatial and temporal dimensions (σ = 2) prior to CSD computation.

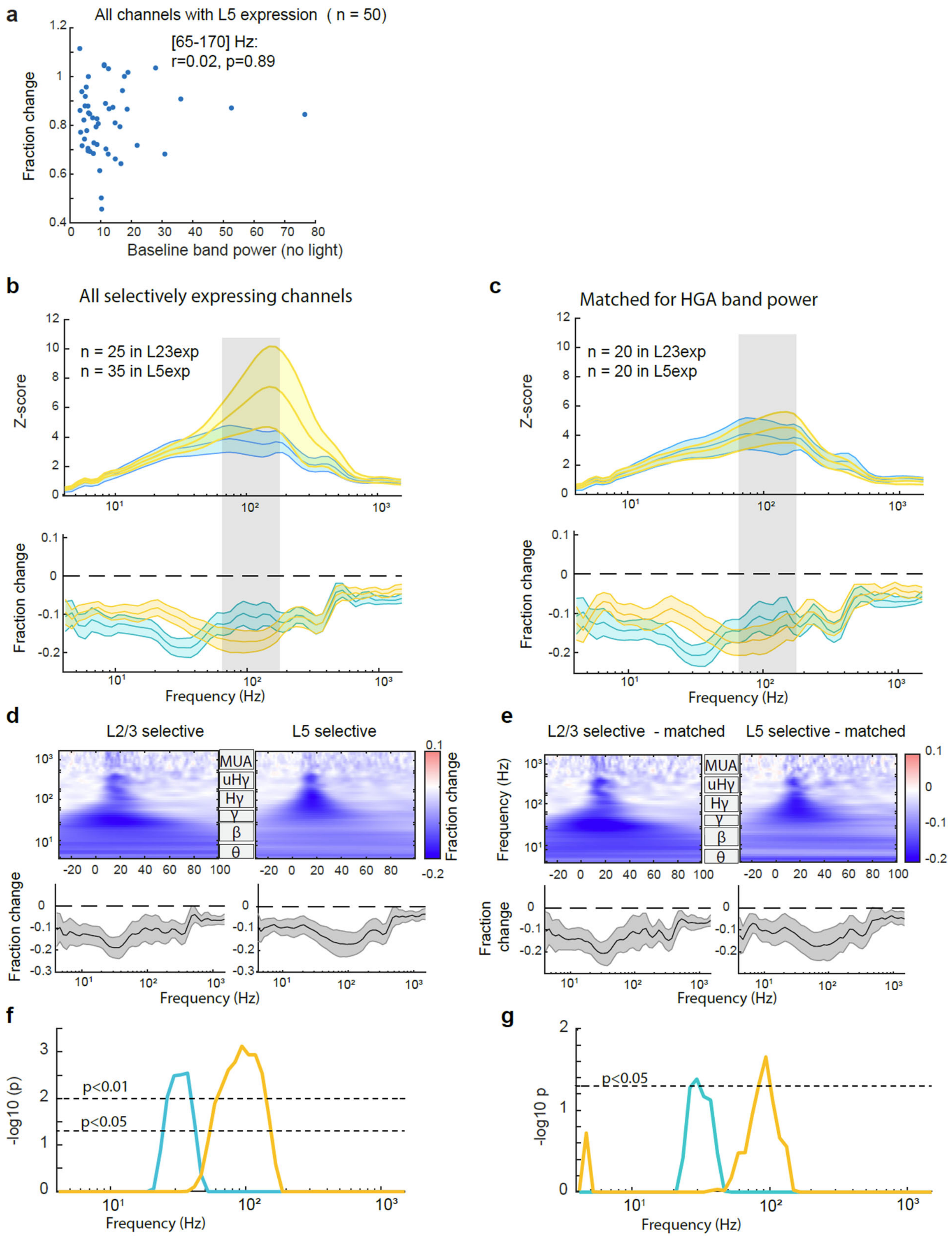

a
All channels with L5 expression (n = 50)
[65-170] Hz:
r=0.02, p=0.89
Fraction change
Baseline band power (no light)
b
All selectively expressing channels
n = 25 in L23exp
n = 35 in L5exp
Z-score
Fraction change
Frequency (Hz)
c
Matched for HGA band power
n = 20 in L23exp
n = 20 in L5exp
Z-score
Fraction change
Frequency (Hz)
d
L2/3 selective
L5 selective
MUA
uHγ
Hγ
γ
β
θ
Fraction change
Frequency (Hz)
e
L2/3 selective - matched
L5 selective - matched
Frequency (Hz)
Fraction change
Frequency (Hz)
f
-log10 (p)
p<0.01
p<0.05
Frequency (Hz)
g
-log10 p
p<0.05
Frequency (Hz)

Supplementary Figure 4. Frequency-specific suppression cannot be explained by baseline (no-light) evoked-response differences.

a, Absence of correlation between the no-light, evoked, band power and the fraction change (65-170Hz; 10 ms peak evoked activity, Spearman rank correlation coefficient). We found similar results across all frequency bands (all -025<r<0.20 , p>0.05, N = 50 peak-channels with L5 expression). **b**, Overlaid sensory evoked response (Z-scored) for L2/3-selective and L5-selective groups of peak-channels, all channels included (groups defined in Figure 3). Note the stronger high-gamma response in the L5-expressing channels. **c**, Same as (b) after sub-sampling the channels to match the response in the high-gamma band between the two groups (random-search subsampling of both groups until they are undistinguishable, i.e. p>0.5 with a two-samples t-test). Note the similarity of spectral evoked response in the two subsampled groups of selected channels. **d-e**, spectrogram of fraction change (top row) and quantifications across peak-channels (bottom row) for each group of selective expression (left and right). (**d)**, all peak channels included. (**e)**, matched peak channels only. Shading is 95% CI across peak-channels (see n in figure panels (b) and (c). **f-g**, Frequency-selective suppression across the spectrum in the L5-selective and L2/3 selective groups of peak channels. **(f)**, all channels included. **(g)**, matched peak channels only.
